# Supplementary material for: Utilizing a multi-stage transition model for analysing child stunting in two urban slum settlements of Nairobi: A longitudinal analysis, 2011-2014
Source: PLoS One. 2024 Feb 26;19(2):e0272684. doi: 10.1371/journal.pone.0272684 (PMC10896550; doi:10.1371/journal.pone.0272684)
Supplement: Attachment — Submitted filename: Response to Reviewers.pdf [file pone.0272684.s001.pdf]

PONE-D-22-20839: A multi-state transition model for child stunting in two urban slum settlements of Nairobi: a longitudinal analysis, 2011-2014

Replies to Reviewers' Comments

RESPONSE TO EDITOR

Comment: Comments to Author.

Comments. Thank you for submitting your manuscript to PLOS ONE. After careful consideration, we feel that it has merit but does not fully meet PLOS ONE's publication criteria as it currently stands. Therefore, we invite you to submit a revised version of the manuscript that addresses the points raised during the review process.

Please submit your revised manuscript by Sep 14 2023 11:59PM. If you will need more time than this to complete your revisions, please reply to this message or contact the journal office at plosone@plos.org. When you're ready to submit your revision, log on to https://www.editorialmanager.com/pone/ and select the 'Submissions Needing Revision' folder to locate your manuscript file.

Please include the following items when submitting your revised manuscript: A rebuttal letter that responds to each point raised by the academic editor and reviewer(s). You should upload this letter as a separate file labeled 'Response to Reviewers'. A marked-up copy of your manuscript that highlights changes made to the original version. You should upload this as a separate file labeled 'Revised Manuscript with Track Changes'. An unmarked version of your revised paper without tracked changes. You should upload this as a separate file labeled 'Manuscript'. If you would like to make changes to your financial disclosure, please include your updated statement in your cover letter. Guidelines for re-submitting your figure files are available below the reviewer comments at the end of this letter.

Response. We are grateful to for the opportunity to response to the reviewers' comments and revise the manuscript.

Point-by-point responses have been provided to address all comments.

Journal's requirement

Comment: Comment #1

Comments. *Please ensure that your manuscript meets PLOS ONE's style requirements, including those for file naming. The PLOS ONE style templates can be found at https://journals.plos.org/plosone/s/file?id=wjVg/PLOSOne_formatting_sample_main_body.pdf and https://journals.plos.org/plosone/s/file?id=ba62/PLOSOne_formatting_sample_title_authors_affiliations.pdf*

Response. We have used the PLOS ONE styple template in the revised submission.

Comment: Comment #2

Comments. *Please update your submission to use the PLOS LaTeX template. The template and more information on our requirements for LaTeX submissions can be found at http://journals.plos.org/plosone/s/latex.*

Response. The revised manuscript has been produced using the PLOS LaTeX template as suggested.

Comment: Comment #3

Comments. *Thank you for stating the following in the Acknowledgments Section of your manuscript:*

We sincerely acknowledge those who contributed to the establishment of the NUHDSS, especially Alex Ezeh and Eliya Zulu. We also acknowledge funding support for the NUHDSS received from a number of donors including the Rockefeller Foundation (USA), the Wellcome Trust (UK), the William and Flora Hewlett Foundation (USA), Comic Relief (UK), the Swedish International Development Cooperation (SIDA) and the Bill and Melinda Gates Foundation (USA).

We note that you have provided funding information that is not currently declared in your Funding Statement. However, funding information should not appear in the Acknowledgments section or other areas of your manuscript. We will only publish funding information present in the Funding Statement section of the online submission form. Please remove any funding-related text from the manuscript and let us know how you would like to update your Funding Statement. Currently, your Funding Statement reads as follows:

The author(s) received no specific funding for this work.

Please include your amended statements within your cover letter; we will change the online submission form on your behalf.

Response. We thank you for the comment and suggestion. Indeed, no specific funding was received for this research. The acknowledgement of the funding support for the NUHDSS has been removed from the acknowledgement section since this is not a direct support for this present research work.

Comment: Comment #4

Comments. We note that you have indicated that data from this study are available upon request. PLOS only allows data to be available upon request if there are legal or ethical restrictions on sharing data publicly. For more information on unacceptable data access restrictions, please see http://journals.plos.org/plosone/s/data-availability#loc-unacceptable-data-access-restrictions.

In your revised cover letter, please address the following prompts:

a) If there are ethical or legal restrictions on sharing a de-identified data set, please explain them in detail (e.g., data contain potentially sensitive information, data are owned by a third-party organization, etc.) and who has imposed them (e.g., an ethics committee). Please also provide contact information for a data access committee, ethics committee, or other institutional body to which data requests may be sent.

b) If there are no restrictions, please upload the minimal anonymized data set necessary to replicate your study findings as either Supporting Information files or to a stable, public repository and provide us with the relevant URLs, DOIs, or accession numbers. For a list of acceptable repositories, please see http://journals.plos.org/plosone/s/data-availability#loc-recommended-repositories.

We will update your Data Availability statement on your behalf to reflect the information you provide.

Response. The data is owned by the African Population and Health Research Center (APHRC) and is available upon request through the Center's micro data portal `http://microdataportal.aphrc.org/index.php/catalog`. Our data availability statement should be revised as follows:

> " The data used for this research is owned by the African Population and Health Research Center (APHRC) and is available upon request through the Center's microdata portal which can be assessed using this link: `http://microdataportal.aphrc.org/index.php/catalog` "

Comment: Comment #5

Comments. One of the noted authors is a group or consortium Nairobi Urban Health and Demographic Surveillance System. In addition to naming the author group, please list the individual authors and affiliations within this group in the acknowledgments section of your manuscript. Please also indicate clearly a lead author for this group along with a contact email address.

Response. The NUHDSS is not a group or a consortium but a surveillance system. We are unable to list all the authors who have contributed to the system. We have therefore removed this from the authors list.

Comment: Comment #6

Comments. Your ethics statement should only appear in the Methods section of your manuscript. If your ethics statement is written in any section besides the Methods, please move it to the Methods section and delete it from any other section. Please ensure that your ethics statement is included in your manuscript, as the ethics statement entered into the online submission form will not be published alongside your manuscript.

Response. We have included the following ethics statement in the data description section of the methodology:

> "The NUHDSS and IVP study were granted ethical clearance by the Ethical Review Board of the Kenya Medical Research Institute (KEMRI). Anonymized dataset was obtained from the African Population and Health Research Center (APHRC) microdata portal. Thus, there is no risk of harm to the study participants."

Comment: Comment #7

Comments. Please review your reference list to ensure that it is complete and correct. If you have cited papers that have been retracted, please include the rationale for doing so in the manuscript text, or remove these references and replace them with relevant current references. Any changes to the reference list should be mentioned in the rebuttal letter that accompanies your revised manuscript. If you need to cite a retracted article, indicate the article's retracted status in the References list and also include a citation and full reference for the retraction notice.

Response. The reference list has been reviewed and updated accordingly.

RESPONSE TO REVIEWER #1

Comment: General comment

Comments. I read through the paper, and I must admit that this paper is a critical piece of missing information that the field of nutrition, public health and stunting requires: on 'mechanism of injury (pathophysiology and causation in public health" on stunting as a marker of 'disadvantage'. I read the paper with this lens. It covers all the ground well, including the methodology, which is fascinating as this type of analysis is only done in computer science fields–so this is an innovative and creative way of analysing the problem, and then quantifying it in a manner that makes public health sense.

Response. We appreciate the positive feedback on our manuscript.

RESPONSE TO REVIEWER #2

Comment: General comment

Comments. Thank you for this very interesting and important piece of work. The problem of stunting is a considerable challenge and your efforts to establish some of its determinants among vulnerable people in Kenya should be lauded. There are a number of issues with the manuscript that needs addressing - please see my specific comments below.

Response. We are grateful to the reviewer for the comprehensive review of the manuscript and for providing very insightful comments to reshape and improve the manuscript. We have attempted to address the comments and suggestions to the best of our ability.

Comment: Introduction #1

Comments. The description of stunting as it pertains to Height-for-Age Z-scores also appears in the Methods, where I feel it is more suited. Please consider changing "It is measured by...are considered stunted." to "Stunting is defined as a child who has low height for his or her age.", and then providing the technical details pertaining to Z-scores and standard deviations to the Methods.

Response. Thanks to the reviewer for this comment. This has been addressed. The technical details are in the methods, and the sentence has been rephrased as suggested.

Comment: Introduction #2

Comments. "It can further lead to irreversible brain damage." The cited paper by Cooper et al. does not say this.

Response. The cited paper was comprehensively examined, and we agree with the reviewer's position. Thus, this statement has been deleted.

Comment: Introduction #3

Comments. Sentence beginning with "The past two decades have seen..." - It is not clear what is meant by "with twice as much declining rates". Please rephrase this for clarity.

Response. Thanks once again to the reviewer for pointing this out. This sentence has been rephrased and updated with a corresponding reference for better clarity. This was rephrased to

> "The past two decades have seen a considerable reduction in the number of stunted children in Latin America and the Caribbean, where in particular, stunting has declined twice as quickly as in Africa from 2000 to 2016"

Comment: Introduction #4

Comments. Sentence beginning with "Since stunting mostly..." - Please change "complementary feed" to either "complementary feeding" or "complementary food".

Response. As suggested, "complementary feed" has been changed to "complementary feeding".

Comment: Introduction #5

Comments. Challenges around complementary feeding and breastfeeding (largely qualitative considerations) as well as MMS (largely high costs) need to be highlighted here for balance. This also supports in illustrating later findings around feeding, maternal education, and wealth.

Response. Challenges around complementary feeding and breastfeeding as well as MMS have been addressed for balance and relevant literature has been cited. This now reads as:

> "However, access to nutritious foods for complementary feeding might be limited due to financial constraints, geographic location, or availability of diverse food options, affecting the quality of the child's diet. Furthermore challenges such as the lack of support, maternal employment, and family misconceptions and/or influences about breastfeeding might lead to early cessation of exclusive breastfeeding or reduced frequency of breastfeeding. Also, Multiple micronutrient supplements can be expensive, making them inaccessible for vulnerable populations in low-income settings. This cost factor can limit the effectiveness of interventions aimed at improving nutritional status. Also, ensuring a consistent and reliable supply of multiple micronutrient supplements, especially in remote or resource-constrained areas, can be difficult due to logistical issues and infrastructure limitations. "

Comment: Introduction: #6

Comments. Sentence beginning with "Lack of healthcare, sanitation..." - Please change "maternal education, urban / rural residency" to "maternal education, and urban or rural residency".

Response. This change has been made.

Comment: Introduction #7

Comments. Sentence beginning with "Studies conducted in..." - Please consider changing the archaic "environs" to "environments".

Response. This change has been made.

Comment: Introduction #8

Comments. Sentence beginning with "However, stunting prevalence is..." - Please change "low socioeconomic status (SES) households" to "low socioeconomic status (SES) urban households".

Response. This has been addressed.

Comment: Introduction #9

Comments. Please revise the way findings from references 32 and 33 are presented in the sentence "Among this population, glaring..." Ref 32 makes a very different comparison. i.e., between poor rural and rich urban populations; Ref 33 also makes comparisons around wealth exclusively, not urban/rural setting

Response. Thanks to the reviewer for this comment. The findings have been revised and updated with emphasis on the stunting disparity between children living in urban and rural settings. The reference of Gewa & Nandell, 2011 was more relevant to the statement and hence was kept. This now reads as;

> "Among this population, disparities are observed between rural and urban child dwellers. Specifically, among the older group of children, living in the urban areas, compared with rural areas, was associated with higher odds of underweight in Kenya. "

Comment: Introduction #10

Comments. Sentence beginning with "For instance, between..." - Please change to "For instance" to "In addition". For instance does not make sense in this context as the statement does not follow on from the previous comparative works.

Response. This has been revised accordingly.

Comment: Materials and methods #11

Comments. Sentence beginning with "The NUHDSS is a..." - Please check the spelling of "Koroogcho".

Response. Thanks for highlighting this typo. This has been addressed.

Comment: Materials and methods #12

Comments. Sentence beginning with "Since 2002, the..." - Please remove "nutrition" and "vaccination" from the brackets listing health outcomes. These are exposures and should be listed as such. The "other data" in the next sentence are social determinants of health and could be identified as such, if the authors so choose.

Response. We thank the reviewer for this comment. This has been revised accordingly.

Comment: Materials and methods #13

Comments. Please write out unitless counts below ten; e.g., "across 6 Health and Demographic Surveillance Systems", "at most 3 per child per year". "The outcome has 4 levels". There may be others not listed here.

Response. Thanks to the reviewer for this suggestion. They have been revised.

Comment: Materials and methods #14

Comments. Please specify the month of study commencement in 2010 and the month the study ended in 2014. Does this correspond to the 40 months in the transition probability diagram in Figure 3? If so, please make a statement that the study duration was 40 months.

Response. We have clarified in the data description that the study was conducted from 2010 to 2014, with data collection starting in March 2011 to June 2014. The longest follow-up time for a child in the study was 40 months.

Comment: Materials and methods #15

Comments. Please indicate the ages of children included in the full sample of 3419 children.

Response. We have made the correction that the 3419 were the number of observation times for all the children and not the number of children in the study.

Comment: Materials and methods #16

Comments. Sentence beginning with "Among the various data..." - Please remove "age" from the brackets listing anthropometric measures and list this as a separate dimension.

Response. Thanks to the reviewer for this comment, this has been revised.

Comment: Materials and methods #17

Comments. The subset of 692 children is difficult to reconcile with 2889 observations in Table 1. Please indicate how these children are represented in these tables over time - presumably, children had to have follow-up visits (a maximum of three visits are listed earlier) to provide observations around state transitions; how is it possible that 692 full anthropometric datasets could be obtained if only n=117 children were observed in 2014 (Table 1)?

Response. Table 1 has been revised. Since children entered and exited the study at different times, we have now reported descriptive statistics at the time of entry into the study. Specific changes to the figures have been highlighted in the revised manuscript.

Comment: Materials and methods #18

Comments. The discrepancy between 2889 observations in Table 1 and 2197 observations in Table 2 needs clarification.

Response. As indicated in the earlier responses, these numbers are not the same. Table 1 shows the number of children, and Table 2 represents the number of observed transitions from one state to another at the different observed times.

Comment: Materials and methods #19

Comments. Please justify or provide a reference to substantiate why children aged 0 to 3 years were included. For example, https://www.mattioli1885journals.com/index.php/actabiomedica/article/view/11346 suggests stunting affects development up to the age of 4 years - would it not have been more comprehensive to include a broader age range?

Response. Children between the 0 to 3 years were recruited to allow sufficient time to observe their outcomes before their 5th year. Thus, outcomes of children between 4 and 5 were observed for those with late entry year to the study. Also note that this is a secondary data analysis and therefore we did not have control over age range of the children to be included in the study.

Comment: Materials and methods #20

Comments. Please change "...household socio-economic data was obtained..." to "...household socio-economic data were obtained..."

Response. This has been revised.

Comment: Materials and methods #21

Comments. As mentioned in the Introduction comments, the Methods section does a good job of describing the measurement of stunting using HAZ. Please simplify the Introduction section and using some detail from there in the Methods section - in particular, the description of Z-scores as standard deviations from the median growth standard is missing here.

Response. As suggested more details from the introduction regarding the description of WHO's child growth standards has been included in the methods section. The following statement has therefore been added:

> "As per WHO global child growth standards, children whose HAV scores are 2 standard deviations less than the median growth standard are considered stunted"

Comment: Materials and methods #22

Comments. There is very likely (multi)collinearity between several of the explanatory variables - while it is understood that this does not explicitly change predictive value of single explanatory variables, and acknowledged that the Akaike information criterion was used in the model, this does need to be stated. The reader needs to be aware that many of these variables are correlated; sections in the Discussion section needs to take into account that suggested policy changes for one element may have an influence on many.

Response. We are grateful for the comment. We computed the Cramer's V statistics for all the categorical variables. However, the association between these variables were weak (all values were < 0.2). We have added the following statement to the Statistical Analysis section.

"Before fitting the model, the Cramer's V statistics were computed to assess any potential correlation between the categorical independent variables. All associations between these independent factors were weak (Cramer's V values < 0.2), eliminating any fear of multicolinearity"

Comment: Materials and methods #23

Comments. IMPORTANT: Were ALL explanatory factors measured again at EVERY VISIT where anthropometric information was obtained? Specifically, were 'fixed' characteristics such as maternal education, exclusive breastfeeding, parental marital status measured at every visit? If not, the rationale for investigating back-transition (state improvement) as it pertains to these characteristics does not hold - for example, if maternal post-primary education (measured only once) is correlated with back-transition, there can be no conclusion that $>$increased$<$ maternal education has a role to play in reducing stunting, as the level of education was constant (also while the child was transitioning to poorer states). Furthermore, this approach will likely result in a high chance of spurious associations, many of which may be driven by other collinear factors.

Response. Indeed, the authors can confirm from the data that all explanatory factors measured again at every visit where anthropometric information were obtained. Furthermore, fixed characteristics such as maternal education, exclusive breastfeeding, parental marital status were also measured at every visit.

Comment: Materials and methods #24

Comments. A reference to Figure 1 is needed at the start of the Statistical Analysis section.

Response. A reference to Figure 1 has now been made.

Comment: Materials and methods #25

Comments. The terse mathematical description refers to state space as [uppercase] S initially, then [lowercase] s throughout the rest of the section and, indeed, paper.

Response. The uppercase, S, has been updated to a lowercase letter. This is now consistent throughout the paper.

Comment: Results #26

Comments. *Table 1 shows the percentage of children who transitioned from one stunting state to another. Should this be Table 2?*

Response. Indeed, it should be Table 2. This has been corrected.

Comment: Results #27

Comments. *"Generally, we observed that fewer...also when back transitioning." Please revise this sentence as the meaning is not clear at all.*

Response. This sentence has been re-phrased for clarity. It now reads as

> "Generally, it was observed that the proportion of children transitioning from a normally stunted state to another state decreased. This observation was similarly made for when they transitioned back from other states into a normally stunted state."

Comment: Results #28

Comments. *Transitions that had percentages less than 10% were not considered in the model. These categories still represent upwards of 70 children. Please provide a reference or rationale for this decision. While I do understand that this may be a question of face validity, it is worrying that predictors for the transition we would MOST like to avoid (normal to severe) have not been explored, however rare it may be in the real world.*

Response. We wish to clarify again that the values do not represent the number of children but the number of such transitions in the course of the study. These transitions were not considered because the number of observations was not sufficient to estimate the model parameters for these transitions. We have clarify the statement as follows:

> "Transitions that had percentages less than 10% were not considered in the multi-state modeling due to the small number of observation for these transitions which hampers the estimation of model parameters associated with these transitions."

Comment: Results #29

Comments. *It is not clear why Figure 3 presents transition probabilities for the state transitions representing less than 10%. If these were not included in the model, why were TPs calculated and presented?*

Response. Figure 3 has been revised to include only transitions of interest.

Comment: Results #30

Comments. *"On average, children spend about 2.4 (95% CI [1.1, 5.4]) months...and 2.27 (95% CI [1.1, 4.9]) months, respectively." Where are these values coming from? If these are additional model outputs, please provide them as accompanying or supplementary tables.*

Response. These are the estimated mean sojourn times which we have computed and are not derived from any of the tables. We have clarify this in the revised manuscript which now reads as follows:

> "We also estimated the mean sojourn times from the model. On average, children spend about $2.4(95\%\text{CI}[1.1, 5.4])$ months in normal state before moving to other states. Furthermore, the average times spent in the marginally stunted, moderately stunted, and severely stunted state before transitioning to other states are estimated as 1.7(95% CI $[1, 3]), 2.1(95\%\text{CI}[1.4, 3.2])$, and 2.27 (95% CI $[1.1, 4.9]$) months, respectively."

Comment: Results #31

Comments. *As mentioned previously, please indicate if the 40 months in Figure 3 corresponds to the duration of the study.*

Response. We have clarified in comment #14 that this represents the longest follow-up time in the study.

Comment: Results #32

Comments. *The entire section 3.2.2. (Factors associated with each transition state) is extremely lexically dense and difficult to digest. I would suggest further subdividing these sections with sub-headings - consider adding headings for (1) explanatory child characteristics, (2) explanatory maternal/parental characteristics, (3) explanatory social determinants of health? Alternatively, these results may also be easier to consume in a table, perhaps arranging from the factor posing the greatest to the smallest risk?*

Response. This section has been subdivided into the suggested headings to make it more digestible.

Comment: Results #33

Comments. *It is not clear why Table 4 is not referred to in the section detailing 'Normal to marginally stunted and backwards transitions".*

Response. This table has been referenced at the beginning of the section.

Comment: Discussion and Conclusion #34

Comments. *The first paragraph of this section is largely a repetition of the results. This paragraph would benefit from a discussion of the possible mechanisms underlying these findings.*

Response. The possible mechanisms underlying the findings in that beginning paragraph from the authors' perspective have been included. These additional statements were included:

> "There are conceivable mechanisms that may explain the underlying reasons for the findings presented in this study, and underscore the urgent need for targeted government policies and interventions to address the disparities observed in stunting transitions between the two slum settlements and more broadly, that observed between ethnicities. Viwandani and Korogocho likely exhibit variations in socioeconomic conditions, access to essential resources like clean water, nutritious food, and healthcare services. These disparities might contribute to the differing likelihoods of transitions between stunting states.
>
> Limited access to resources in one settlement could lead to more prolonged stunting states due to inadequate nutrition and healthcare. Furthermore, environmental factors, such as living conditions, sanitation, and exposure to contaminants, can play a significant role in child health. Variations in environmental conditions between the two settlements might influence the progression of stunting states. For instance, poorer sanitation conditions could lead to higher rates of infections, exacerbating stunting.
>
> Disparities in healthcare access and health awareness can impact the likelihood of transitioning out of stunting states. If one settlement or ethnic group has better access to healthcare services and nutrition education, children might have a higher chance of transitioning to a healthier state. Also, differences in government policies and interventions targeting these slum settlements and ethnic groups can also play a role. Variances in the effectiveness and reach of these policies might contribute to the observed disparities in stunting transitions"

Comment: Discussion and Conclusion #35

Comments. *"This is further confirmed by Brown, et. al." This reference could not be found; it is also noted that it is very old. Please try to find a more recent paper to substantiate this statement. If not available, please provide the full citation for Brown 1999 in the reference list.*

Response. We agree that the current reference may be old, and have provided a more recent reference in Bork & Diallo 2017 to corroborate the preceding statement.

Comment: Discussion and Conclusion #36

Comments. *The findings of the study by Rakotomanana et. al. that are presented are not the same as the present study. The present study finds rapid state transitions at 0-5 months as juxtaposed with older ages; Rakotomanana and colleagues found that stunting increased with age.*

Response. Thanks, this reference has been deleted from the list of references that supported the idea of rapid state transitions juxtaposed with older ages. Darteh et. al, 2014 and Takele, Zewortir & Ndanguza 2019 rather justify this assertion and have been left on that list.

Comment: Discussion and Conclusion #37

Comments. *It is not clear what the findings of the study by Emily et. al. are - children in their second year of life have a higher likelihood to be underweight and stunted as compared to what?*

Response. Thanks for the comment. Emily et. al. inferred via cross-sectional studies that in their second year of life, children in Western Kenya have a higher likelihood of being underweight and stunted when compared with the first year (0–12 months). This has been updated in the manuscript for clarity.

Comment: Discussion and Conclusion #38

Comments. *Please change "food insecured" and food secured" to "food insecure" and "food secure", respectively.*

Response. These changes have been made.

Comment: Discussion and Conclusion #39

Comments. *Sentence beginning with "The effect of parent marital status..." - Please change "largely significant" to something like "notable". This finding is not significant if it is not statistically significant.*

Response. These changes have been made. “largely significant” has been changed to “notable”.

Comment: *Discussion and Conclusion #40*

Comments. IMPORTANT: From "Regarding the effect of mother's educational status...odds of child stunting in Malawi, Tanzania, and Zimbabwe." Back-transitioning is NOT the same as never having been stunted. The findings from other studies suggesting that maternal education results in lower odds of being stunted indicates a protective association of maternal education. As stated before, back-transition associated with maternal education can ONLY show the latter as a protective factor if maternal education was measured every time anthropometric measures were taken, and maternal education was increasing as stunting was decreasing.

Response. Thanks for the comment. Indeed, as we have previously established in addressing the other reviewer's comments the authors can confirm from the data that all explanatory factors were measured again at every visit where anthropometric information was obtained. Fixed characteristics such as maternal education, exclusive breastfeeding, and parental marital status were also measured at every visit. Also, the authors would like to emphasize that we have not established in any part of the manuscript that back-transitioning is the same as never having been stunted.

Comment: Discussion and Conclusion #41

Comments. I find "menace" to be a strange, emotive and redundant addition to "stunting". I'll defer to the editor as this is a style choice, but would prefer if it was not framed in this way.

Response. Menace has been excluded from stunting in the conclusion.

Comment: Discussion and Conclusion #42

Comments. IMPORTANT: It is fairly disorienting to paper where no reflections regarding the strengths and limitations of the study are presented as part of the Discussion. Please consider adding this to the manuscript.

Response. Thanks to the reviewer for highlighting this. As suggested we have included in the later part of the discussion the strengths and potential limitations of this study. This reads as

> “Taking into account the study's strengths and potential limitations is crucial. Firstly, as far as we know, this study is pioneering in its utilization of a comprehensive multi-state transition modeling

approach, enabling the exploration of intricate transitions between various child stunting states and their associated factors. Moreover, this research was conducted within two urban slum settlements in Nairobi, shedding light on a marginalized and often neglected population. Additionally, the inclusion of participants' ethnicity recognizes the sway of cultural elements on child stunting transitions. Nevertheless, it's important to emphasize that the accuracy of the study's outcomes hinges on the quality of the measured data. The existence of measurement errors could potentially impact the conclusions drawn. Moreover, the findings might not be universally applicable to other settings due to the distinct characteristics of the studied slum settlements and ethnic groups. Lastly, it's important to acknowledge the potential presence of unmeasured confounding variables. Socioeconomic indicators and caregiving practices, unaccounted for in this study, could potentially influence the observed associations."
