# Supplementary material for: Utilizing a multi-stage transition model for analysing child stunting in two urban slum settlements of Nairobi: A longitudinal analysis, 2011-2014
Source: PLoS One. 2024 Feb 26;19(2):e0272684. doi: 10.1371/journal.pone.0272684 (PMC10896550; doi:10.1371/journal.pone.0272684)
Supplement: Attachment — Submitted filename: Response to Reviewers 2.pdf [file pone.0272684.s002.pdf]

PONE-D-22-20839R1: A multi-state transition model for child stunting in two urban slum settlements of Nairobi: a longitudinal analysis, 2011-2014

Replies to Reviewers' Comments

RESPONSE TO EDITOR

Comment: Comments to Author.

Comments. Thank you for submitting your manuscript to PLOS ONE. After careful consideration, we feel that it has merit but does not fully meet PLOS ONE's publication criteria as it currently stands. Therefore, we invite you to submit a revised version of the manuscript that addresses the points raised during the review process.

Please submit your revised manuscript by Jan 19 2024 11:59PM. If you will need more time than this to complete your revisions, please reply to this message or contact the journal office atplosone@plos.org. When you're ready to submit your revision, log on to https://www.editorialmanager.com/pone/ and select the 'Submissions Needing Revision' folder to locate your manuscript file.

Please include the following items when submitting your revised manuscript: A rebuttal letter that responds to each point raised by the academic editor and reviewer(s). You should upload this letter as a separate file labeled 'Response to Reviewers'. A marked-up copy of your manuscript that highlights changes made to the original version. You should upload this as a separate file labeled 'Revised Manuscript with Track Changes'. An unmarked version of your revised paper without tracked changes. You should upload this as a separate file labeled 'Manuscript'. If you would like to make changes to your financial disclosure, please include your updated statement in your cover letter. Guidelines for resubmitting your figure files are available below the reviewer comments at the end of this letter

Response. We are grateful to for the opportunity once again to respond to the reviewers' comments and revise the manuscript. Point-by-point responses have been provided to address all comments.

Journal's requirement

Comment: Comment #1

Comments. Please review your reference list to ensure that it is complete and correct. If you have cited papers that have been retracted, please include the rationale for doing so in the manuscript text, or remove these references and replace them with relevant current references. Any changes to the reference list should be mentioned in the rebuttal letter that accompanies your revised manuscript. If you need to cite a retracted article, indicate the article's retracted status in the References list and also include a citation and full reference for the retraction notice.

Response. Thanks to the editor for highlighting this. All references have been comprehensively reviewed and none of those have been retracted. All references cited are very relevant to this manuscript. Thanks once again.

RESPONSE TO REVIEWER #2

Comment: General comment

Comments. Dear authors, Thank you very much for engaging so constructively with the previous round of feedback, this is much appreciated. The paper is looking great and is, in my view, more clear and accessible for the reader following your revisions. Please see a few additional comments below:

Response. We are grateful to the reviewer for acknowledging the improvement of the manuscript in the first round of revisions, and for providing additional insightful comments to further improve the manuscript. We have attempted to address the comments and suggestions to the best of our ability.

Comment: Introduction #1(previously comment #1)

Comments. Thank you very much for the revision of the description around stunting in the Introduction and Materials and methods sections. Two small follow-up comments in the Materials and Methods section-there is a typo in Line 124 (please change 'HAV' to 'HAZ'); also, please consider adding a sentence explaining why there is a 'marginal' stunting category even though it does not meet the strict WHO definition of stunting (<2 SDs below the median). My suggestion for the latter would be to add the following: '...and severely stunted (HAZ <-3). While marginal stunting does not fall into the strict definition of stunting, it cannot be ignored as it represents a barrier to thriving (Tredoux, C., Dawes A., Mattes, M. (2022) Thrive by Five Index 2021 Technical Report (Revised July 2022). University of Cape Town and Innovation Edge, Cape Town.) In addition to the stunting state...'

Response. Thanks to the reviewer for highlighting the typo and the included suggestion. The typo has been fixed and the suggested sentence on marginal stunting has been included.

Comment: Comment Materials and methods #2 (previously comment #19):

Comments. Thank you very much for the clarification that children between the 0 to 3 years were recruited to allow sufficient time to observe their outcomes before their 5th year and that, therefore, outcomes of children between 4 and 5 were observed for those with late entry year to the study. Would it be possible to add a sentence to support the reader with this understanding?

Response. We have added the following statement to the manuscript to offer readers with this understanding.

> "Consequently, outcomes were assessed for some children during follow-up when they reached 40 months, specifically for those who entered the study at 2 to 3 years of age."

Comment: Comment Materials and methods #3 (previously comment #22):

Comments. The authors are thanked for considering the comment regarding collinearity and for further statistical calculations to estimate this. Without the numeric values, however, it is not clear now weak these associations are -please note some sources consider a Cramér's V of > 0.10 moderate and > 0.15 strong (Dai, J., Teng, L., Zhao, L., & Zou, H. (2021). The combined analgesic effect of pregabalin and morphine in the treatment of pancreatic cancer pain, a retrospective study. Cancer Medicine, 10(5), 1738–1744. https://doi.org/10.1002/cam4.3779). It is acknowledged that other sources do define $V < 0.2$ as weak; in the presence of this conflicting advice, it is recommended that exact values are presented and the reference defining these as 'weak' cited.

Response. As advised, we have included Cramer's V values for some variables that potentially could be collinear. The following statement has therefore been added to the manuscript:

> For example, the Cramer's V value for the relationship between food security and wealth quartile was 0.149, between access to sanitation and wealth status was 0.054, and between access to sanitation and food security was 0.088.

The reference "Kotrlik JW, Williams HA, Jabor MK. Reporting and Interpreting Effect Size in Quantitative Agricultural Education Research. Journal of Agricultural Education. 2011; 52(1):132-142." has also been added.

Comment: Comment Results #4 (previously comment #27):

Comments. *The authors are thanked for revising this section of text for clarity. It is not apparent that the revision is reflecting the previously intended meaning (interpreted as stating that very few children skipped a consecutive state of severity as they transitioned, either forward or backward, through states). It appears from the reduction of Figure 1 to Figure 2 in the revised manuscript as though these events were less than 10% in each case and therefore not considered in the model. If this is not the case, it is up to the authors whether they want to retain the more general current statement, or reconsider.*

Response. Thanks to the reviewer for this comment. We have slightly changed the sentence to enhance clarity. This now reads

> ...Transitions that had percentages less than 10% were not considered in the multi-state modeling due to the small sample size for these transitions which hampers the estimation of model parameters associated with these transitions. Thus, some of the hypothesized transitions were not estimated and the schematic diagram for the 4-state transition shown in Figure 1 reduces to Figure 2

Comment: Comment Results #5, Line 282-284

Comments. *'The risk of back transitioning from the moderately stunted to marginally stunted state for children in moderately food secured and severely food secured households are, respectively, 2.9 and 1.7 times the risk for children in food secured households' does not say the same as the Discussion: '...we observed that children living in households that experienced moderate to severe food insecurity were at an increased risk to transition into a moderately stunted state from a marginally stunted state, relative to those living in food secure households.' Please revise lines 282-284 for clarity – it is not clear what 'moderately food secured' and 'severely food secured' means in relation to 'food secured'.*

Response. Thanks very much to the reviewer for highlighting this. This was an obvious error. The lines 282-284 have now been revised, and is consistent with the discussion. It now reads

> The risk of back transitioning from the moderately stunted to marginally stunted state for children living in moderately food insecure and severely food insecure households are, respectively, 2.9 and 1.7 times the risk for children in food secure households.

Comment: Comment Results #6, Line 293-295:

Comments. *'...when in a marginally stunted state, children whose mothers have attained primary or post primary education are, respectively, 2.85 times and 3.6 more likely to transition into a moderately stunted state compared to children with a less than primary educated mothers.' does not say the same as the Discussion: '...the results indicated that children in a moderately stunted state were more likely to back-transition to marginally stunted state if they have mothers with primary and post-primary education. This finding is consistent with a study by Abuya et. al. [35], who observed that children born to primary educated mothers were at a significantly lower odds of being stunted relative to mothers with no primary education.' Please revise lines 293-295, which currently implies that children of mothers with primary+ education are at higher risk of transitioning from marginally to moderately stunted.*

Response. Thanks once again for this important comment. The lines 293-295 have now been revised according to the results, is consistent with the statements made in the discussion, and reflects the ideas in Abuya et. al [35]. It now reads

> In addition, when in a moderately stunted state, children whose mothers have attained primary or post primary education are, respectively, 3.02 times and 2.71 more likely to transition into a marginally stunted state compared to children with a less than primary educated mothers.

RESPONSE TO REVIEWER #3

Comment: General comment

Comments. *Thank you for submitting this great piece of work for publication in this journal. You have chosen a topic of immense public health importance, not only to Kenyan society, but to the entire tropical environment. The communication level and use of English language is excellent. I believe that this manuscript is suitable for publication in this journal but I also have a few comments and observations which require further attention from the authors.*

Response. We are grateful to the reviewer for the comprehensively reviewing the manuscript and for the positive feedback. We have attempted to clarify(when necessary) and address the comments and suggestions.

Comment: Comment #1

Comments. *I am not sure if the title of the article (A multi-state transition model for child stunting in two urban slum settlements of Nairobi: a longitudinal analysis, 2011-2014) correctly describes the contents. As it is, it suggests that the main outputs of this work are some models developed to predict or quantify*

stunting while in reality, the authors used modeling techniques to measure and classify stunting into 4 degrees of severity. I believe that the authors can improve the understanding of the title by making some adjustment to it.

Response. We have revised the title as follows:

> "Utilizing a multi-stage transition model for analysing child stunting in two urban slum settlements of Nairobi: a longitudinal analysis, 2011-2014."

Comment: Comment #2

Comments. *I am also a bit concerned about the selection of the subset of 692 children as study participants because they were the ones with complete anthropometric data, and whose household socioeconomic data were obtained from the NUHDSS. This set of children may not be representative of all children from these slums. The reasons why they have complete anthropometric data and household socioeconomic data may also make them different from the other children without complete information. Could the authors also performed statistical analysis of key potentially confounding factors between these children with complete information (research participants) and those with incomplete information (excluded from participation) to see how similar or different they are? Again related to study design, how were the 'factors associated with transition between stunting states' determined or chosen?*

Response. The use of the word 'subset' must have been misleading. The 692 children were randomly selected from the surveillance for the IVP study which measured the anthropometric outcomes. We have therefore revised the statement as follows:

> "In this study, data on 692 children with complete anthropometric information, obtained from the IVP study, and whose household socio-economic data were obtained from the NUHDSS were used for the analysis."

Comment: Comment #3

Comments. *In your discussion, I expected to see how the authors discuss the positive or negative effects (if any) between one stunting state to another in order to highlight the significance of these transitions. Is it feasible to add something on that?*

Response. Thanks to the reviewer for seeking clarification. Respectfully, we would like to emphasize that, in the discussion, negative or positive effects between stunting states were quantified in terms of the "risk"(negative) and probability of back transitioning from a less desired state to a more desired stunting state "positive". For instance, instance, the discussion captured that,

> ...children from Viwandani, when in a marginally stunted state, were less likely to move into a moderately stunted state in comparison to Korogocho children ("**positive**"). For the effect of ethnicity on child stunting, we found that moderately stunted children living in Luo, Kamba and Luhya households were at a relatively lower risk of transitioning into normal state when compared to those living in Kikuyu households ("**also positive**"). There was also a lower risk for them to transition from marginally stunted to normal state ("**positive**").

Furthermore, for example, it was also discussed that

> ...Observing food security effect on stunting transitions, we observed that children living in households that experienced moderate to severe food insecurity were at an increased risk ("**negative**") to transition into a moderately stunted state from a marginally stunted state, relative to those living in food secure households.

Several literature were cited, and conceivable mechanisms underlying these "negative" or "positive transitions" were highlighted. Thanks once again for clarifying.

Comment: Comment #4

Comments. Finally, I also expect the authors to emphasize on the need for early screening of stunting before the age of 6 months since children between the age of 0 - 5 months are more likely to transitioned from poorer stunting states to better statuses.

Response. Thanks to the reviewer for this comment. As requested, we have included statements in the discussion highlighting the need for early stunting screening before the age of 6 months. These additional statements have been included in the discussion and highlighted.

> ...Given the observation that children aged 0-5 months are more likely to rapidly transition among the four stunting states compared to the other age groups, early screening during this period is essential, as it allows for the implementation of targeted and responsive interventions. Whether through nutritional supplementation,

breastfeeding support, or health education for caregivers, these interventions can effectively support a child's growth trajectory, minimize long-term consequences, and contribute to overall health and well-being
